# An Integrated In Vitro and In Silico Investigation of the Bioactive Properties of Wild *Glycyrrhiza glabra* var. *glandulifera*

**DOI:** 10.1007/s11130-025-01304-z

**Published:** 2025-02-10

**Authors:** Hamit Emre Kızıl, Sibel Ulcay, Yavuz Ekincioğlu, Hatice Öğütçü, Güleray Ağar

**Affiliations:** 1 Vocational School of Health Services, Department of Medical Services and Techniques, Bayburt University, Bayburt, 69010 Türkiye; 2 Faculty of Agriculture, Department of Field Corps, Kırşehir Ahi Evran University, Kırşehir, 40100 Türkiye; 3 Polatlı Faculty of Science and Letters, Department of Biology, Hacı Bayram Veli University, Ankara, 06100 Türkiye; 4 Faculty of Science, Department of Biology, Ataturk University, Erzurum, 25240 Türkiye

**Keywords:** *Glycyrrhiza glabra* var. *glandulifera*, Glycyrrhizic acid, Antimicrobial activities, Cytotoxicity, Braf, Molecular docking

## Abstract

This investigation examined methanol extracts obtained from the whole plant (encompassing all organs except roots), flowers, and fruits of wild *Glycyrrhiza glabra* var. *glandulifera*, a liquorice cultivar indigenous to the Cappadocia region of Türkiye, constituting the first comprehensive in vitro and in silico analysis of this species within the region. Anatomical identification revealed distinctive structural characteristics, including thick-walled epidermal cells in the stem, organized clusters of collenchyma cells, and well-defined sclerenchyma layers encompassing the vascular bundles. The petiole exhibited a characteristic heart-shaped morphology containing three vascular bundles, while foliar analysis demonstrated the presence of stomata on both adaxial and abaxial surfaces, accompanied by a bifacial mesophyll arrangement. The extracts demonstrated remarkable antimicrobial efficacy against both Gram-positive and Gram-negative bacteria, as well as various fungal species. Furthermore, significant cytotoxic activity was observed against non-small cell lung cancer (H460), with the whole plant extract exhibiting IC_50_ values of 117.8 µg/mL (24 h) and 105.6 µg/mL (48 h). Flower extracts demonstrated IC_50_ values of 116.8 µg/mL (24 h) and 112.7 µg/mL (48 h), while fruit extracts displayed superior potency with IC_50_ values of 104.4 µg/mL (24 h) and 63.09 µg/mL (48 h). Molecular docking analyses, conducted utilizing AutoDock Tools 1.5.6 and BIOVIA Discovery Studio Visualizer 4.5, revealed significant protein-ligand interactions, yielding optimal binding energy and inhibition constant values of -9.57 kcal/mol and 96.92 nM, respectively. The RMSD values were calculated based on structural variations between ligand-protein complexes during re-docking procedures. These findings underscore the substantial biological potential of *Glycyrrhiza glabra* var. *glandulifera* from Cappadocia, necessitating further comprehensive investigation.

**Supplementary Information:**

The online version contains supplementary material available at 10.1007/s11130-025-01304-z.

## Introduction

Liquorice has maintained its global prominence throughout centuries, being extensively valued for its distinctive aromatic properties and remarkable sweetening characteristics. *Glycyrrhiza glabra* L., exhibiting widespread distribution across Asia, Europe, Africa, and Australia [1], represents a herbaceous species attaining heights of 1 m, distinguished by its compound leaves comprising 9–17 leaflets, inflorescences featuring pale whitish-blue to purple flowers (0.8–1.2 cm), and characteristic oblong fruit pods (2–3 cm) [2]. The species demonstrates substantial therapeutic, pharmaceutical, and industrial applications [3]. Within the Glycyrrhiza genus, encompassing 30 species, G. glabra is particularly noteworthy for its extensive array of bioactive constituents, including glycyrrhizin, glabridin, glabrene, glabrol, licoflavonol, and various other compounds [4]. Contemporary research has extensively investigated its therapeutic efficacy, with particular emphasis on cancer treatment, antimicrobial properties, ulcer management, and anti-inflammatory capabilities [5]. A 2023 investigation identified liquiritigenin as a potential SARS-CoV-2 inhibitory agent [6], while a comprehensive 2022 study analyzing geographical variants demonstrated significant pharmacological properties through cytotoxicity assessments and diverse cellular experimental protocols [7]. Non-small cell lung cancer (NSCLC), constituting 75–80% of lung cancer cases, continues to exhibit poor long-term survival outcomes despite advancements in therapeutic interventions [8]. BRAF mutations, observed in 1–3% of NSCLC cases, play a fundamental role in early tumorigenesis and are categorized into V600E and non-V600E variants [9, 10]. While plant-derived compounds have demonstrated potential in BRAF kinase modulation, the underlying molecular mechanisms remain to be fully elucidated. The emergence of in silico methodologies has transformed drug discovery paradigms, offering cost-effective alternatives to conventional testing approaches and addressing significant challenges in drug development, where approximately 40% of candidates fail due to toxicological considerations [11].

This investigation aims to elucidate the biological properties of wild *Glycyrrhiza glabra* var. *glandulifera* indigenous to Türkiye’s Cappadocia region and evaluate the in silico anticancer potential of glycyrrhizic acid targeting B-Raf Kinase in NSCLC, thereby contributing to the expanding knowledge base regarding this therapeutically significant plant species. This study aims to investigate the biological properties of wild *Glycyrrhiza glabra* var. *glandulifera* from Türkiye’s Cappadocia region and examine the in silico anticancer potential of glycyrrhizic acid targeting B-Raf Kinase in NSCLC, contributing to the growing body of knowledge about this valuable medicinal plant.

## Materials and Methods

This section is presented as Supplementary Material (SM).

## Results and Discussion

### Anatomical Identification

Structural analysis of plant specimens constitutes a fundamental methodology in both taxonomic identification and pathological diagnosis, particularly for species exhibiting morphological complexities. Such anatomical insights prove instrumental in molecular phylogenetic investigations, where structural characteristics facilitate the elucidation of evolutionary patterns. Despite the considerable significance of anatomical studies, it is noteworthy that *G. glabra* var. *glandulifera* lacks comprehensive anatomical characterization. Morphological examination revealed that the stem exhibited an irregular circular configuration with distinctive indentations. Cross-sectional analyses demonstrated thick-walled epidermal cells (circular and rectangular) encompassed by a substantial cuticle layer, characterized by the presence of both simple non-glandular and peltate glandular trichomes. The restricted cortical region comprised 10–12 stratified layers of clustered collenchyma cells. Vascular bundles demonstrated dense arrangement, surrounded by 8–10 layers of sclerenchymatous tissue. The phloem region exhibited considerable expansion, with a distinctive 3–4 layered cambial zone separating the xylem and phloem tissues. The expansive pith region consisted of heterogeneous circular parenchymatous cells, interspersed with secretory elements (Fig. S1A-B-C). The characteristic heart-shaped petiole exhibited uniseriate epidermal cells (oval or rectangular) featuring both non-glandular and glandular (peltate and capitate) trichomes. The anatomical organization included 3–4 layers of parenchymatous tissue subjacent to the epidermis, with circular parenchyma cells encompassing the vascular bundles (Fig. S1D-E-F). Foliar cross-sections revealed circular and quadrangular epidermal cells with peltate glandular and simple non-glandular trichomes. The leaves demonstrated amphistomatic organization with anomocytic stomatal complexes (3–5 subsidiary cells) distributed on both surfaces (Fig. S1H-I). The prominent mid-vein region contained a single vascular bundle. The bifacial mesophyll organization comprised 2–3 layers of rectangular palisade parenchyma and 3–4 layers of spongy parenchyma cells occupying equivalent spatial distributions (Fig. S1G) (Table 1). Based on these morphoanatomical characteristics, taxonomist Assoc. Prof. Sibel ULCAY confirmed the specimen’s identity as *Glycyrrhiza glabra* var. *glandulifera* indigenous to the Cappadocia region (Herbarium number: 384).

### Antimicrobial Assay Results

The antimicrobial efficacy of methanol extracts derived from *G. glabra* var. *glandulifera* whole plant (comprising all organs except roots), flowers, and fruits was evaluated against selected pathogenic bacterial and fungal strains, utilizing standard antibiotics (SXT25: sulfamethoxazole, AMP10: ampicillin, K30: kanamycin) and antifungal agents (NYS100: nystatin) as comparative controls. The extracts exhibited variable antimicrobial activity, demonstrating inhibition zones ranging from 20 to 40 mm against the tested microorganisms.

**Table 1:** Anatomical measurements of various cells

Plant organ		Width/diameter (µm) Mean ± SE	Length (µm) Mean ± SE
Stem	Epidermis cells	9.98 ± 0.76	16.53 ± 2.58
	Cortex parenchyma cell	19.17 ± 1.70	
	Collenchyma	12.89 ± 1.06	
	Trachea	36.59 ± 2.18	
	Pith parenchyma cell	22.01 ± 1.79	
Leaf	Upper epidermis	12.32 ± 0.69	29.45 ± 1.65
	Lower epidermis	16.85 ± 1.09	12.70 ± 0.87
	Palisade parenchyma	21.74 ± 1.33	56.95 ± 3.89
	Spongy parenchyma	14.89 ± 0.92	
	Trachea diameter	15.05 ± 0.96	
	Lower stomata	20.16 ± 0.62	29.79 ± 0.57
	Upper stomata	24.06 ± 0.86	31.25 ± 0.85
Petiole	Epidermis	11.62 ± 0.64	29.26 ± 1.97
	Sclerenchyma	10.62 ± 0.45	
	Parenchyma	46.67 ± 2.86	
	Collenchyma	16.73 ± 1.70	
	Trachea	26.65 ± 1.22	

SE: standard error

The whole plant, flower and fruit extracts demonstrated moderate to high antimicrobial efficacy (20 mm to 27 mm). The whole plant methanol extract showed highest activity against *Salmonella typhi* (35 mm), exceeding standard antibiotic effectiveness. It also showed superior activity against *Pseudomonas aeroginosa* (23 mm), outperforming all standard antibiotics. Against *Staphylococcus epidermidis* and *Bacillus cereus*, the extract produced inhibition zones of 21 mm, comparable to standard antibiotics. Lower inhibition zones were observed for *Staphylococcus aureus* and *Proteus vulgaris* (20 mm). Against *Proteus vulgaris*, the extract showed similar effectiveness to AMC (20 mm) while surpassing AMP (17 mm) and SXT (19 mm). Additionally, the whole plant extract demonstrated antifungal activity against *Candida albicans* equivalent to NYS antifungal reagent (Fig. S1). The flower extract of *G. glabra* var. *glandulifera* exhibited highest inhibition against *P. aeroginosa* (35 mm), exceeding all standard antibiotics. The second largest inhibition zone was observed against *S. epidermis* (31 mm), also surpassing standard antibiotics. Against *P. vulgaris* and *S. typhi*, the extract formed zones of 26 mm and 25 mm respectively, both larger than standard antibiotic zones. The extract also showed activity against *S. aureus* (21 mm) and *B. cereus* (23 mm), though not exceeding standard antibiotics. Additionally, it demonstrated antifungal activity against *C. albicans* (25 mm), surpassing NYS antifungal reagent. The fruit extract demonstrated superior antimicrobial properties (20–40 mm) compared to both whole plant and flower extracts. It showed strongest activity against *S. epidermidis* (40 mm), significantly exceeding all standard antibiotics. *S. epidermis* is an opportunistic pathogen primarily associated with skin and nosocomial infections. Against *S. typhi* pathogen, which can cause various clinical symptoms from asymptomatic to severe typhoid-like syndromes, the extract formed a 35 mm inhibition zone, surpassing all standard antibiotics. The extract also demonstrated strong activity against *B. cereus* and *P. aeruginosa* (both 30 mm), exceeding standard antibiotic effectiveness. While showing relatively smaller inhibition against *P. vulgaris* (21 mm), it matched K30 and exceeded three other standard antibiotics. The extract also showed enhanced antifungal activity against *C. albicans* (21 mm), surpassing NYS100 (Table 2).

**Table 2:** Antimicrobial activity of extracts and standard reagents (diameter of zone of inhibition in mm)

Microorganisms (µg/ml)	Whole plant	Flower	Fruit	AMP10	SXT25	AMC30	K30	NYS 100
*S. epidermis* Gr (+)	21	31	40	26	25	27	25	-
*S. aureus* Gr (+)	20	21	20	30	24	30	25	N
*B. cereus* Gr (+)	21	23	30	23	25	20	28	N
*P. aeroginosa* Gr (-)	23	35	30	8	18	15	14	N
*S. typhi* Gr (-)	27	25	35	11	17	19	20	N
*P. vulgaris* Gr (-)	20	26	21	17	19	20	21	N
*C. albicans* (Fungi)	20	25	21	N	N	N	N	20

*N* not tried, *H* high activity, *I* intermediate activity, *L* low activity. *Standard reagents: *SXT25* (sulfamethoxazole); *AMP10* (ampicillin); *NYS100* (nystatin); *K30* (kanamycin); *AMC30* (amoxicillin)

### Cytotoxicity Assay Results

H460 cells were subjected to treatment with methanol extracts derived from *G. glabra* var. *glandulifera* whole plant, flowers, and fruits at concentrations ranging from 12.5 to 200 µg/mL over 24 and 48-hour exposure periods. The whole plant extract demonstrated statistically significant cytotoxic activity (*p* < 0.05) at 200 µg/mL following 24-hour exposure, yielding an IC_50_ value of 117.8 µg/mL. Prolonged exposure (48 h) resulted in significant cytotoxicity across a broader concentration range (25–200 µg/mL, *p* < 0.05), with a corresponding IC_50_ value of 105.6 µg/mL. The flower extract exhibited significant cytotoxic effects against H460 cells at both 200 and 100 µg/mL concentrations following 24-hour treatment, demonstrating an IC_50_ value of 116.8 µg/mL. Extended exposure to 48 h revealed cytotoxic activity exclusively at 200 µg/mL concentration, with an observed IC_50_ value of 112.7 µg/mL.The fruit extract demonstrated particularly notable cytotoxic efficacy (*p* < 0.05) against H460 cells at both 200 and 100 µg/mL concentrations across both treatment durations. The temporal analysis revealed IC_50_ values of 104.4 µg/mL at 24 h, with enhanced potency observed at 48 h as evidenced by a reduced IC_50_ value of 63.09 µg/mL (Table 3).

**Table 3:** 24. and 48. hours % viability results of *G. Glabra* var. *Glandulifera* whole plant, flower and fruit methanol extracts treatment in H460 (NSCLC) cell line

*Glycyrrhiza glabra* var. *glandulifera* whole plant methanol exctract groups	24th hour	48th hour
	Mean ± SD	Mean ± SD
Control	100 **±** 4.44	100 **±** 2.32
200 µg/mL	16.404 **±** 3.11*	9.460 **±** 1.71*
100 µg/mL	98.977 **±** 1.22	62.970 **±** 1.30*
50 µg/mL	102.248 **±** 1.52	89.726 **±** 2.06*
25 µg/mL	106.439 **±** 1.23	91.866 **±** 2.76*
12.5 µg/mL	108.074 **±** 3.42	93.043 **±** 1.26
***Glycyrrhiza glabra *** **va** **r. ** ***glandulifera*** **flower methanol exctract groups**	**24th hour**	**48th hour**
	**Mean ± SD**	**Mean ± SD**
Control	100 **±** 2.79	100 **±** 0.75
200 µg/mL	36.495 **±** 6,64*	57.493 **±** 3.31*
100 µg/mL	91.081 **±** 3,23*	88.462 **±** 1.49
50 µg/mL	95.589 **±** 7,79	97.571 **±** 2.18
25 µg/mL	98.725 **±** 3,39	100.364 **±** 0.55
12.5 µg/mL	101.078 **±** 1,34	100.121 **±** 3.55
***Glycyrrhiza glabra *** **var. ** ***glandulifera*** **fruit methanol exctract groups**	**24th hour**	**48th hour**
	**Mean ± SD**	**Mean ± SD**
Control	100 **±** 1.10	100 **±** 1.91
200 µg/mL	10.685 **±** 0.13*	3.415 **±** 0.20*
100 µg/mL	69.918 **±** 0.18*	13.639 **±** 3.60*
50 µg/mL	93.419 **±** 2.97	85.446 **±** 4.20
25 µg/mL	99.686 **±** 1.37	107.577 **±** 3.42
12.5 µg/mL	112.638 **±** 2.39	111.426 **±** 1.30

Mean: the average of the test results performed with 3 repetitions, SD: standard deviation, % Cell viability: WST-8 (CVDK-8 kit) results, Control: Untreated cells, *p < 0.05 is significant

### Structure Optimization

The optimization of molecular structure represents a critical parameter in the accurate determination of a compound’s physicochemical and biological characteristics. In this investigation, molecular geometry optimization was performed utilizing density functional theory (DFT) methodology implemented through Gaussian 09 computational software. The resultant optimized structural configuration of the glycyrrhizic acid molecule is illustrated (Fig. S1). Table 4 presents selected geometric parameters, including bond lengths, bond angles, and dihedral angles, computed at the B3LYP/6-311 + + G (d, p) functional level of theory. The computational analysis yielded optimized geometric parameters that demonstrate strong concordance with systematic investigations of hydrogen-bond and Carbon-Carbon bond geometries derived from crystallographic data (as documented in the Cambridge Structural Database) and existing literature [12–14].

**Table 4:** Optimized geometrical parameters of glycyrrhizic acid molecule

Bond length (Å)		Bond angle (^o^)		Dihedral angle (^o^)	
C50-O52	1.35322	C50-O52-H114	110.83447	C51-C50-O52-H114	-175.81701
C50-C13	1.53830	C50-C13-O14	113.51629	C50-C13-O14-C15	87.19462
O10-H64	0.96242	O10-H64-C9	109.12328	O10-H64-C9-C8	-82.36230
C8-O7	1.42699	C8-O7-C6	116.05522	C8-O7-C6-C53	-179.78784
O7-C6	1.39831	O7-C6-O5	107.55856	O7-C6-O5-C4	34.07480
C4-C2	1.51587	C4-C2-O1	120.90743	O5-C4-C2-O1	-168.02149
O58-H120	0.96636	C57-O58-H120	107.95930	C55-C57-O58-H120	175.61133
C29-O30	1.22355	C31-C29-O30	123.78543	C24-C31-C29-O30	-146.73627
C49-C26	1.55815	C49-C26-C48	107.53995	C49-C26-C48-C47	57.41918
C44-H103	1.09599	C45-C44-H103	108.56028	C45-C44-H103-C46	127.68610

### Molecular Docking Research

Molecular docking methodology represents an essential component in pharmaceutical design and development, facilitating the comprehensive analysis of binding affinities and molecular interactions between ligands and their target proteins. BRAF inhibitors constitute a therapeutic class capable of attenuating or suppressing the progression of metastatic melanoma, characterized by malignant dissemination from the primary site to distant anatomical locations [15]. The crystallographic structure of the BRAF kinase domain in its activated conformation (PDB accession code: 4FK3) (www.rcsb.org) was utilized as the target structure for this analytical approach. Active site determination within the investigated protein was accomplished through POCASA V1.1 implementation, complemented by established literature protocols [39, 16, 17]. Docking calculation validation was achieved through systematic re-docking procedures. The ligand-protein complex underwent re-docking analysis, with root-mean-square deviation (RMSD) values being calculated to establish docking precision [18]. RMSD determination was executed utilizing the following mathematical expression to quantify the mean spatial deviation between atomic positions in reference and target structural conformations:

1 $$\:RMDS=\:\sqrt{\frac{1}{N}\:\sum\:_{i=1}^{N}{\left({r}_{i}^{ref}-{r}_{i}^{target}\right)}^{2}}\:$$

Molecular docking analyses were executed utilizing Auto-Dock Vina 1.1.2, implementing grid box parameters in accordance with methodological protocols established by Trott and Olson [39]. The molecular docking preparation protocol encompassed the removal of cofactors and water molecules, followed by the incorporation of polar hydrogen atoms and Kollman charge assignments utilizing Auto-Dock Tools 1.5.6. The grid box parameters were precisely defined with dimensional specifications of 80 × 60 × 70 Å and centralized coordinates of x = 4.449, y = 49.537, and z = 26.405. Grid spacing was standardized at 0.375 Å throughout the analysis. The computational outcomes yielding minimal binding energy values and corresponding inhibitory constants are comprehensively presented in Table 5.

**Table 5:** Binding energies and inhibition constant of glycyrrhizic acid (C_42_H_62_O_16_) with 4FK3 (*Nanomolar, **Micromolar, *** Millimolar)

Ligand	Protein	Mode	Binding Energy (kcal/mol)	Inhibition Constant (Ki)
C_42_H_62_O_16_	4FK3	1	-9.57	96.92 nM*
		2	-7.50	3.17 μm**
		3	-7.40	3.75 μm
		4	-6.55	-
		5	-6.72	11.96 μm
		6	-5.63	74.70 μm
		7	-4.79	308.97 μm
		8	-4.65	393.68 mM
		9	-4.46	-
		10	-3.72	1.89 mM***

In this investigation, molecular docking methodology was employed to elucidate inhibition constant values, binding energies, and root-mean-square deviation (RMSD) parameters. A direct correlation exists between protein docking scores and binding energy values, where increasingly negative binding energy values correspond to elevated docking scores. The ligand demonstrated binding energy values ranging from − 9.57 to -3.72 kcal/mol with the selected protein target.

The RMSD value, serving as an indicator of ligand binding precision within the protein’s active region, was calculated as 23.0625, derived from structural differences between ligand-protein complexes obtained during re-docking procedures. This elevated RMSD value indicates suboptimal conformational alignment between the ligand and the protein’s active site.

Analysis of the molecular interactions between C_42_H_62_O_16_ and 4FK3 revealed several key residues participating in binding interactions: GLN530, CYS532, TRP531, PHE583, ARG462, SER465, and LYS578 (Fig. S4). Specifically, GLN530, CYS532, and SER465 residues established three conventional hydrogen bonds with the ligand’s hydroxyl moieties, while ARG462 and LYS578 residues formed two carbon hydrogen bonds with carbonyl groups. Additionally, TRP531 and PHE583 residues demonstrated pi-sigma, alkyl, and pi-alkyl interactions with the ligand’s phenyl ring structure.

Initial anatomical investigations of *G. glabra* var. *glandulifera* revealed stem characteristics analogous to *G. iconica*, specifically regarding lined epidermis and subepidermal collenchyma arrangement [19]. The stem exhibited secretory cells characteristic of Fabaceae family members [20], while the petiole demonstrated a heart-shaped configuration comparable to *Trigonella coerulescens* subsp. *kemerensis*. The taxon exhibited amphistomatic leaf organization, with stomatal distribution on both foliar surfaces, consistent with previous observations in *G. iconica* [21]. The foliar epidermis of *G. glabra* var. *glandulifera* demonstrated both simple trichomes and peltate glandular structures, corroborating previous observations by Öztürk et al. [22].

Abbas et al. [1] evaluated the antimicrobial efficacy of methanolic extract and various fractions (n-butanol, ethyl acetate, chloroform, and n-hexane) derived from *G. glabra* roots sourced from Faisalabad, Pakistan. Antimicrobial assessment utilized disc diffusion methodology and minimum inhibitory concentration (MIC) determination against diverse microorganisms, including *P. multocida*, *E. coli*, *B. subtilis*, *S. aureus*, and fungal strains *A. flavus*, *A. niger*, and *R. solani*, demonstrating moderate antimicrobial potency.

Literature regarding endemic Turkish liquorice species remains limited. A comparative analysis of *Glycyrrhiza* samples from the Türkiye-Syria border evaluated antimicrobial and antioxidant properties, revealing superior antimicrobial efficacy of root extracts against Gram-negative bacteria compared to foliar extracts, with antioxidant activity ranging from 4.2 to 2196 mg/mL. Additionally, significant cytotoxicity against human cancer cells was observed, with IC_50_ values ranging from 16.28 to 171.04 mg/mL [23].

Extensive research has demonstrated the anticancer potential of *G. glabra* extracts and isolated metabolites. 18-β-glycyrrhetinic and glycyrrhizic acids have exhibited anticancer effects against cervical, uterine, ovarian, gastric, hepatic, and hematological malignancies. Glycyrrhetic acid derivatives have demonstrated suppressive effects on breast cancer cell lines (MCF-7, MDA-MB-231) [24]. Investigations utilizing *G. glabra* root extract against oral cancer cell lines yielded an IC_50_ value of 43.6 µg/ml [25].

Non-small-cell lung cancer (NSCLC) represents a significant global health burden, with squamous cell carcinoma (SCC) and adenocarcinoma (ADC) constituting predominant subtypes. Glycyrrhetinic acid has demonstrated selective inhibition of thromboxane synthase in lung cancer cells (A549, NCI-H460) while preserving normal lung cell viability. Lectochalcone-A (Lic-A), a principal phenolic constituent, exhibits significant antioxidant properties and antiproliferative effects, suppressing phosphorylated Akt and consequently inhibiting migration and invasion in lung cancer cells [28].

XTT colorimetric analyses revealed significant cytotoxicity of glycyrrhetinic acid (GA) against A549 and H460 cells, with IC_50_ values of approximately 78 and 62 µg/mL, respectively. Mechanistic studies demonstrated GA-induced apoptosis in H460 cells via PKC α/βII inhibition and JNK activation [26].

Comparative analysis of nine geographically distinct *G. glabra* root samples demonstrated variable cytotoxicity against HaCaT, A549, and HepG2 cell lines, as assessed by MTT assay [27]. Glycyrrhizin’s antitumor effects in lung adenocarcinoma appear partially mediated through thromboxane A synthase modulation [28]. Licorice-induced G0/G1 cell cycle arrest correlates with CDK4-Cyclin D1 complex suppression and elevated PD-L1 expression, while in vivo studies demonstrate enhanced antigen presentation and CD8 + T cell infiltration [29]. Glycyrrhizin suppresses tumor growth in PDX models through HMGB1 reduction, potentially via JAK/STAT pathway inhibition [30].

BRAF inhibitors represent FDA-approved therapeutic options for BRAF-positive malignancies [31]. Our molecular docking analyses identified key residues (ARG691, PRO705, GLU713, ARG709, SER706, ASP702) within the 4FK3 protein, yielding binding energy of -4.56 kcal/mol, RMSD of 22.08, and inhibition constant of 455.31 µM. These comprehensive findings suggest potential therapeutic applications of G. glabra extracts in NSCLC treatment, warranting further investigation.

## Conclusion

This investigation examined the pharmacological characteristics of *G. glabra* var. *glandulifera* indigenous to the Cappadocia region of Türkiye. The existing literature demonstrates a notable paucity of research regarding the pharmacological properties of *Glycyrrhiza glabra* var. *glandulifera*, a distinct variety within the *Glycyrrhiza* species complex. Our investigation represents the first comprehensive analysis of this variety within the Cappadocia region.

Environmental parameters, including climatic conditions, edaphic factors, altitudinal variation, and stress conditions, are recognized as significant modulators of medicinal properties in plant species, even among conspecific populations across different geographical locations. While traditional investigations of licorice have predominantly focused on root material, reflecting conventional consumption patterns, our study implements a more comprehensive analytical approach through simultaneous examination of multiple plant organs, with particular emphasis on floral and fruit tissues. Our findings revealed differential antimicrobial activity among extracts, with fruit extracts demonstrating superior activity, followed by flower and whole plant (excluding root) extracts. Each extract exhibited distinct IC_50_ values against the H460 lung cancer cell line. This organ-specific approach represents a significant departure from previous research paradigms that primarily emphasized root material. Molecular docking analyses investigating C42H62O16 binding to 4FK3 identified key active site residues including GLN530, CYS532, TRP531, PHE583, ARG462, SER465, and LYS578. The computational analysis yielded optimal binding energy of -9.57 kcal/mol and inhibition constant of 96.92 nM, with an RMSD value of 23.0625. We may conclude that this study will serve as a valuable guide for future research. This research establishes a foundation for developing potential pharmaceutical ingredients, particularly focusing on glycyrrhizin’s promise as a lung cancer drug candidate.

## Electronic Supplementary Material

Below is the link to the electronic supplementary material.

Supplementary Material 1

## Data Availability

No datasets were generated or analysed during the current study.
