# Supplementary material for: An Integrated In Vitro and In Silico Investigation of the Bioactive Properties of Wild Glycyrrhiza glabra var. glandulifera
Source: Plant Foods Hum Nutr. 2025 Feb 10;80(1):64. doi: 10.1007/s11130-025-01304-z (PMC11811428; doi:10.1007/s11130-025-01304-z)
Supplement: Supplementary file 1 — Supplementary Material 1 [file 11130_2025_1304_MOESM1_ESM.docx]

**Electronic Supplementary Material**

**An Integrated *In Vitro* and *In Silico* Investigation of the Bioactive Properties of Wild *Glycyrrhiza glabra* var. *Glandulifera***

**Hamit Emre KIZIL^1,*^, Sibel ULCAY^2^, Yavuz EKİNCİOĞLU^1^, Hatice ÖĞÜTÇÜ^2,3^, Güleray AĞAR^4^**

^1^Bayburt University, Vocational School of Health Services, Department of Medical Services and Techniques, 69010, Bayburt, Türkiye

^2^Kırsehir Ahi Evran University, Faculty of Agriculture, Department of Field Corps, 40100 Kırsehir, Türkiye

^3^Hacı Bayram Veli University, Polatlı Faculty of Science and Letter, Department of Biology, 06100, Ankara, Türkiye

^4^Ataturk University, Faculty of Science, Department of Biology, 25240, Erzurum, Türkiye

* Corresponding author.

E-mail address: ekizil@bayburt.edu.tr (Hamit Emre KIZIL)

**Materials and Methods**

**Collection, Anatomical Identification and Extraction of Samples**

Specimens of *G. glabra* var. *glandulifera* (Fabaceae) were collected from a location 5 kilometers south of Kırşehir (Türkiye) during the 2020 flowering period (May-July). Taxonomic identification was conducted according to Davis's "Flora of Türkiye" [29]. The collected specimens were preserved using two distinct methods: alcohol preservation and herbarium preparation. Plant material (100 g) underwent methanolic extraction utilizing a Soxhlet apparatus (ISOPAD | Heidelberg, Germany) for 72 hours at controlled temperature conditions. The resulting extracts were filtered through Whatman No. 1 filter paper and subsequently concentrated in vacuo at 60°C using a rotary evaporator (Buchi Labortechnik AG | Flawil, Switzerland). Following lyophilization, the extracts were stored at +4°C under dark conditions until analysis [3]. For anatomical investigations, specimens were preserved in 70% ethyl alcohol solution. Manual sectioning was performed on stem, leaf, and petiole tissues. The sections were processed into permanent preparations using the glycerin-gelatin methodology. Additionally, epidermal sections were obtained from both adaxial and abaxial leaf surfaces. Quantitative analysis involved 25 replicate measurements per parameter. Microscopic documentation was accomplished using a SOIF BK500-L microscope equipped with an AmScope FMA050 camera system for both transverse and superficial sections [31].

**Antimicrobial Assay**

The antimicrobial investigation encompassed diverse pathogenic microorganisms, including three Gram-positive bacteria (*Staphylococcus epidermidis* ATCC12228, *Staphylococcus aureus* ATCC 29213, and *Bacillus cereus* RSKK863), three Gram-negative bacteria (*Pseudomonas aeruginosa* ATCC27853, *Salmonella typhi* H NCTC9018394, and *Proteus vulgaris* RSKK96026), and one yeast strain (*Candida albicans* Y-1200-NIH). Antimicrobial screening was conducted using the well-diffusion methodology as previously described [32]. Dimethyl sulfoxide (DMSO), employed as the solvent control, demonstrated no inherent antimicrobial activity against the test organisms. The experimental protocol involved solubilizing heterocyclic thiosemicarbazone complexes in DMSO (3.5 μg mL−1) and cultivating pathogenic microorganisms in Nutrient Broth agar (10−6 CFU mL−1) for 24 hours at 37°C. The cultures were subsequently homogenized with Mueller-Hinton Agar (MHA) at 45°C and transferred to sterile Petri dishes for solidification. Wells (6 mm diameter) were created in the solidified agar for extract introduction. Following 24-hour incubation at 37°C, inhibition zones were measured and averaged from duplicate experiments. Comparative analysis utilized standard antibiotics including SXT25 (sulfamethoxazole), AMP10 (ampicillin), NYS100 (nystatin), K30 (kanamycin), and AMC30 (amoxicillin) [33].

**Cytotoxicity Assay**

The H460 non-small cell lung cancer cell line was procured from the American Type Culture Collection (ATCC | USA). Cell propagation was conducted in 25 ml flasks (Jet Biofil | Guangzhou, China) using RPMI 1640 medium supplemented with L-glutamine (Gibco™ Thermo Fisher Scientific, USA) and 10% Fetal Bovine Serum (FBS) (Gibco™ | Thermo Fisher Scientific, USA). During cell passage procedures, adherent cells were harvested using trypsin-EDTA solution (Gibco™ | Thermo Fisher Scientific, USA) following PBS (Merck | KGaA Darmstadt, Germany) washing. Cultures were maintained at 37°C in a 5% CO_2_ humidified atmosphere for 24-48 hours. Cell viability was evaluated prior to each passage using trypan blue exclusion assay. Following centrifugation and supernatant removal, cells were stained with trypan blue dye (Merck | KGaA Darmstadt, Germany) and enumerated using a Thoma hemocytometer. The tetrazolium-based cell viability assay employs heterocyclic organic compounds that undergo reduction via mitochondrial electron transfer, resulting in formazan formation and subsequent colorimetric change [34]. This conversion is exclusively observed in metabolically active cells, as non-viable cells lack the requisite reductive capacity [35]. The intensity of formazan production correlates directly with viable cell numbers. The WST-8 assay, utilizing a water-soluble tetrazolium salt, was performed using the CVDK-8 kit (Ecotech Biotechnology® | Erzurum, Türkiye). The resultant orange-colored formazan product was quantified spectrophotometrically using a Multiskan™ GO Microplate Spectrophotometer (Thermo Fisher Scientific, USA) [36].

**Theoretical approach**

The molecular geometry optimization of glycyrrhizic acid (C_42_H_62_O_16_) was executed utilizing Density Functional Theory (DFT) calculations implemented in the Gaussian-09 software package [37]. The calculations were performed at the B3LYP/6-311++G(d,p) level of theory, selected for its documented precision and reliability in molecular structure predictions [38]. Molecular docking simulations were conducted using AutoDock Tools 1.5.6 and AutoDock 4.2.6 [39] to evaluate protein-ligand interactions at specific active sites. The protein binding pocket analysis was performed using the POCASA 1.1 web server. The three-dimensional crystallographic structure of the target receptor was retrieved from the RCSB Protein Data Bank (PDB ID: 4FK3, [www.rcsb.org](http://www.rcsb.org)). Visualization and analysis of protein-ligand interactions were accomplished using BIOVIA Discovery Studio Visualizer 4.5 software [40]. Detailed protocols for the docking calculations are elaborated in the molecular docking methodology section.

**Statistical Analysis**

The cytotoxicity experiments were conducted in triplicate, and results are expressed as mean ± standard deviation (SD). Statistical analyses were performed using GraphPad Prism software (version 8.0.1, United States). The Student's t-test was employed for comparative analysis, with statistical significance established at p<0.05.

**References**

1. Davis PH (ed) (1970) Flora of Turkey and the East Aegean Islands, Vol. 3. Edinburgh University Press, Edinburgh
2. Ulcay S (2022) Anatomy, palynology, seed and leaf micromorphology of Turkish endemic *Allium brevicaule* Boiss. & Balansa and *Allium scorodoprasum* ssp. *rotundum* (L.) Stearn. Acta Biol Crac Ser Bot 64(1):27-38. <https://doi.org/10.24425/abcsb.2022.142049>
3. Ülke E, Hasanoğlu Özkan E, Nartop D, Öğütcü H (2022) New Antimicrobial Polymeric Microspheres Containing Azomethine. J Inorg Organomet Polym Mater 32(10):3971-82. https://doi.org/10.1007/s10904-022-02411-z
4. Nartop D, Özkan EH, Öğütcü H, Yetim NK, Özdemir İ (2024) Novel α-N-heterocyclic thiosemicarbazone complexes: synthesis, characterization, and antimicrobial of properties investigation. RSC Adv 14(40):29308-18. https://doi.org/[10.1039/D4RA04002C](https://doi.org/10.1039/D4RA04002C)
5. Mosmann T (1983) Rapid colorimetric assay for cellular growth and survival: application to proliferation and cytotoxicity assays. J Immunol Methods 65(1-2):55-63. <https://doi.org/10.1016/0022-1759(83)90303-4>
6. Riss TL, Moravec RA (2004) Use of multiple assay endpoints to investigate the effects of incubation time, dose of toxin, and plating density in cell-based cytotoxicity assays. Assay Drug Dev Technol 2(1):51-62. <https://doi.org/10.1089/154065804322966315>
7. Yildirim M, Aksakal E, Ozgeris FB, Ozgeris B, Gormez A (2022) Biological Evaluation of Azide Derivative as Antibacterial and Anticancer Agents. Nat Prod Biotechnol 2(2), 105-113. https://doi.org/10.58465/natprobiotech.2022.11
8. Frisch MJ, Trucks GW, Schlegel HB, Scuseria GE, Robb MA, Cheeseman JR, Scalmani G, Barone V, Mennucci B, Petersson GA et al (2009) Gaussian 09, Revision D.01. Gaussian Inc, Wallingford
9. Ekincioğlu Y (2023) Theoretical Investigation of (E)-1-(2, 4-Dichlorophenyl)-3-[4-(morpholin-4-yl) phenyl] prop-2-en-1-one Molecule As a Possible Potential COVID-19 Drug Candidate: Molecular Docking and DFT Calculations. Russ J Phys Chem 97(13), 57-3067. https://doi.org/10.1134/S0036024423130241
10. Trott O, Olson AJ (2010) AutoDock Vina: improving the speed and accuracy of docking with a new scoring function, efficient optimization, and multithreading. J Comput Chem 31(2), 455-461. https://doi.org/10.1002/jcc.21334
11. Dassault Systèmes BIOVIA (2019) Discovery Studio Modeling Environment, Release 2017. Dassault Systèmes, San Diego


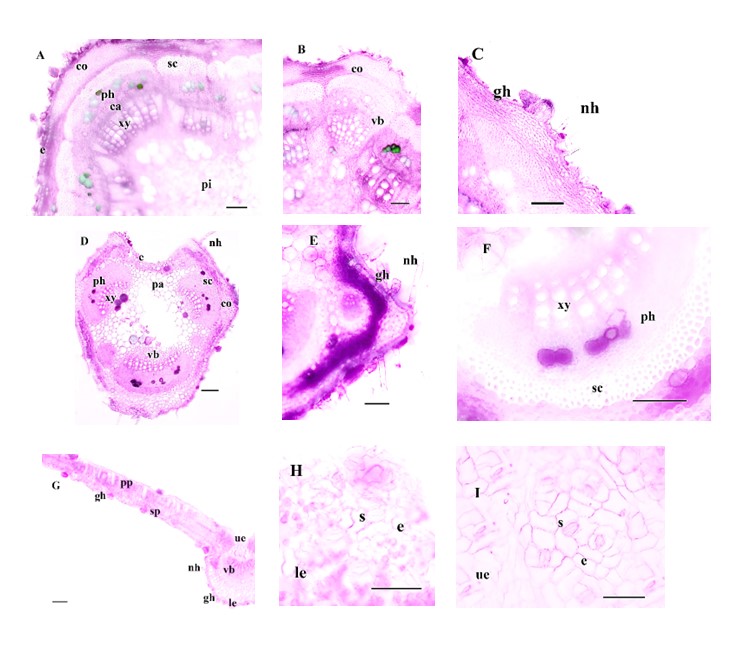


**Fig. S1** *G*. *glabra* var. *glandulifera* A, B, C stem cross-section; D, E, F petiole cross-section; G leaf cross section; H, I leaf superficial sections, ca cambium; co collenchyma; e eperidermis; ; gh glandular hair; le lower epidermis; nh non-glandular hair; ph phloem; xy xylem; pa parenchyma; pi pith; ue upper epidermis; pp palisade parenchyma; s stomata; sc sclerenchyma; sp spongy parenchyma; vb vascular bundless


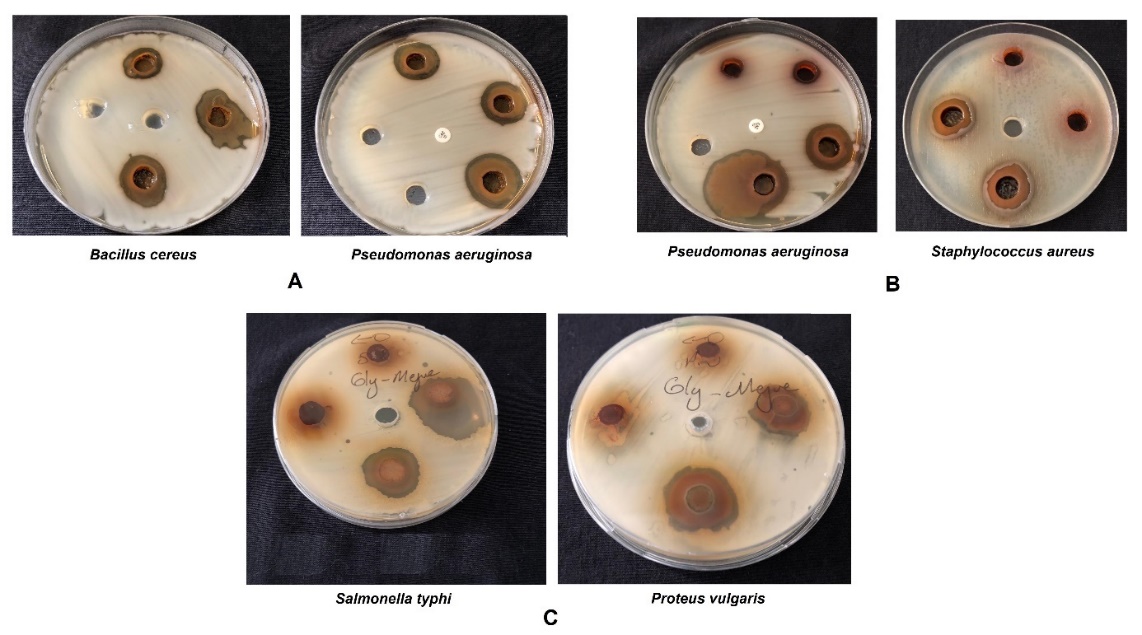


**Fig. S2** Images illustrating the inhibition zones (mm) of various Gram (+) and Gram (–) bacteria: A: whole plant, B: flower, C: fruit.


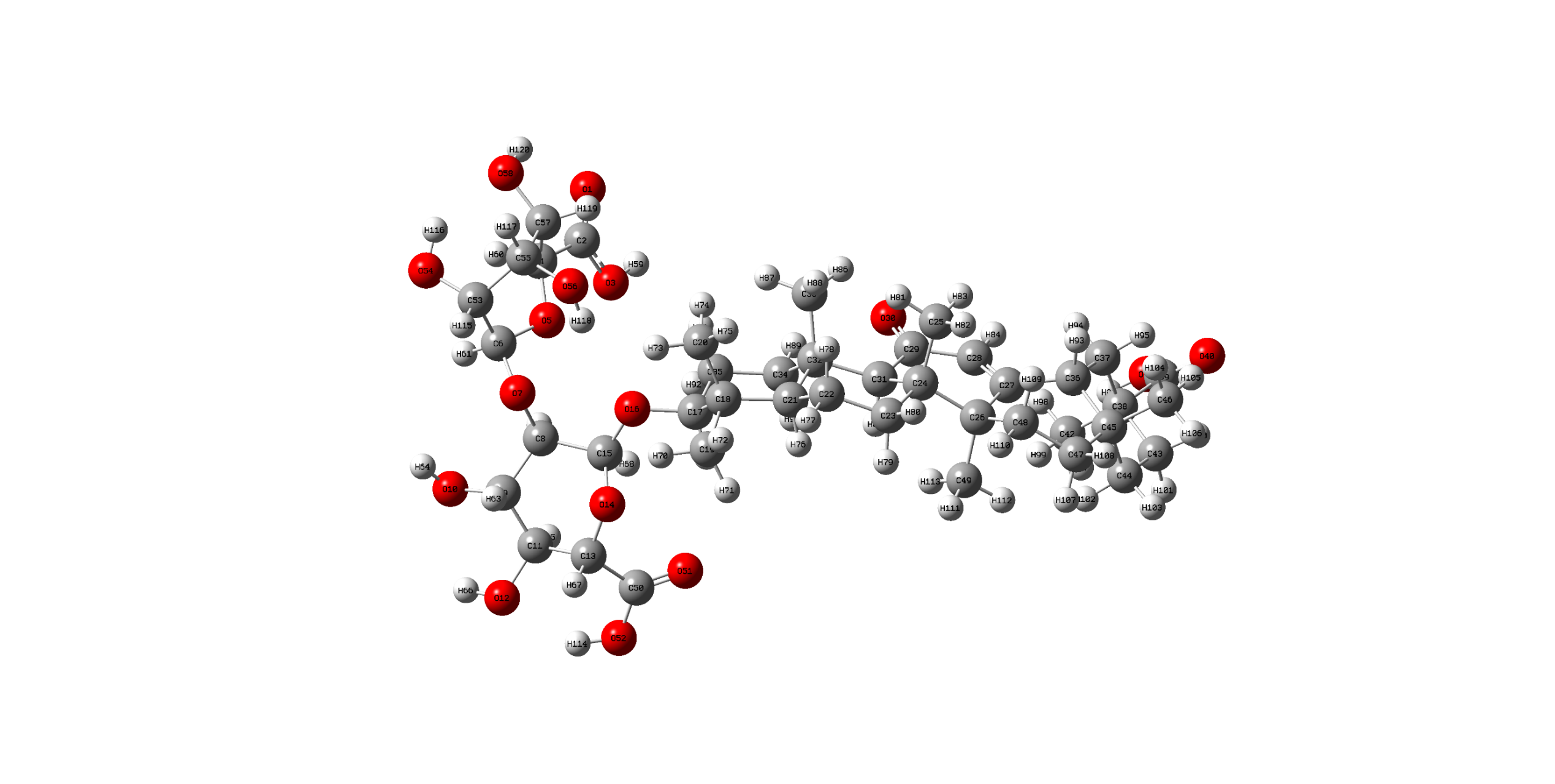


**Fig. S3** The optimized geometry of glycyrrhizic acid molecule


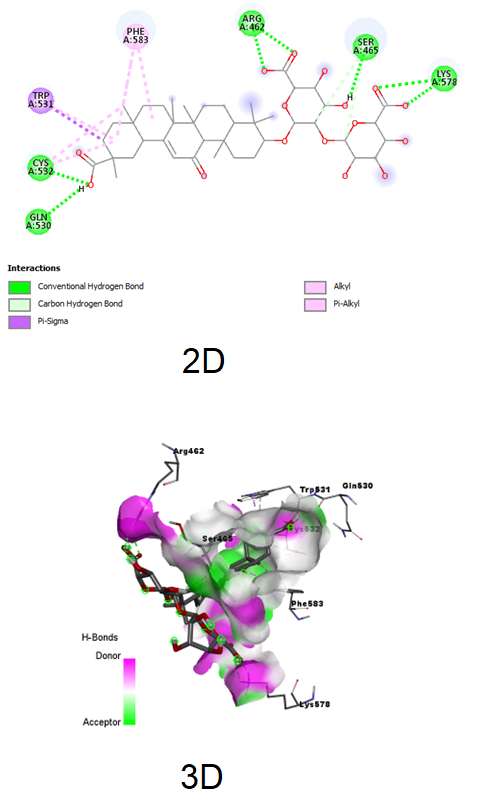


**Fig. S4** The 2D and 3D protein-ligand interaction sites of glycyrrhizic acid with 4FK3.
